# Effect of a six-week times restricted eating intervention on the body composition in early elderly men with overweight

**DOI:** 10.1038/s41598-022-13904-9

**Published:** 2022-06-14

**Authors:** Przemysław Domaszewski, Mariusz Konieczny, Paweł Pakosz, Katarzyna Łukaniszyn-Domaszewska, Wioletta Mikuláková, Ewa Sadowska-Krępa, Steve Anton

**Affiliations:** 1 Department of Health Sciences, Institute of Health Sciences, University of Opole, Katowicka 68, 45-060 Opole, Poland; 2 Faculty of Physical Education and Physiotherapy, Opole University of Technology, Prószkowska 76, 45-068 Opole, Poland; 3 Faculty of Economics and Management, Opole University of Technology, Opole, Poland; 4 Department of Physiotherapy, Faculty of Health Care, University of Presov, Prešov, Slovak Republic; 5 Institute of Sport Sciences, The Jerzy Kukuczka Academy of Physical Education, Katowice, Poland; 6 Department of Aging and Geriatric Research, Institute on Aging, University of Florida, Gainesville, USA

**Keywords:** Risk factors, Diagnostic markers

## Abstract

The main aim of the study was to determine the effectiveness of time-restricted eating (TRE) in reducing body fat and lowering body mass index in early elderly men with overweight (65–74 years). An additional goal was to determine the feasibility of applying TRE for extensive use in elderly men. This study included a group of 46 healthy men (EXP = 23 persons, CON = 23 persons). The six-week intervention in the experimental group involved complete abstinence from food intake for 16 h per day, from 08:00 to 12:00 p.m. After the intervention, the body weight decreased in the EXP group (− 1.92 kg) with a 95% CI (1.14–2.70) compared to the CON group. There was also a decrease in the Visceral fat mass (− 0.64 l) with 95% CI (0.46–0.82) and in the waist circumference (− 3.11 cm) with 95% CI (1.89–4.33) in the EXP group compared to the CON group. The skeletal muscle mass did not change significantly. There was no significant change in the control group, either. The application of TRE in early elderly overweight men resulted in positive changes in body composition and visceral fat. All participants succeed in the prescribed diet plan, which shows that TRE is easy to maintain for early elderly overweight men and may become an essential obesity treatment tool in these age groups.

## Introduction

Natural physiological mechanisms that make it possible to survive starving periods are emblematic for all living creatures. Novel findings indicate that different regimens of fasting may exert positive health effects through multiple pathways, including reducing oxidative stress^1^, optimization of circadian rhythms, restructured gut microbiome, and ketogenesis^2^. Moreover, fasting may modulate cognitive function via various metabolite pathways, including the synthesis and degradation of ketone bodies, butanoate metabolism, pyruvate metabolism, and glycolysis and gluconeogenesis pathways^3^.

Intermittent fasting (IF) is an umbrella term referring to various dietary regimens that cycle between a period of non-fasting and a period of total fasting^4^. In contrast to calorie restriction (CR), the focus of IF regimens is on restricting the time-period in which food is consumed on a daily or ongoing basis. This can result in a reduction in calorie intake, due to the shorter time in which food is consumed^5^. Both aforementioned dietary interventions may result in overall decreased caloric intake, nonetheless this is not integral to intermittent fasting.

In principle, three variations of intermittent fasting are to distinguished: alternate day fasting (ADF), periodic fasting (PF), and time-restricted eating (TRE) (time-restricted feeding (TRF) in animals). In ADF, the subset may consist of 24-h fasts followed by a 24-h eating period that can be done several times a month. Usually, PF characterized a 5/2 strategy when there are 2 fast days mixed into 5 nonrestrictive days. For TRE programs, alternatives include 16-h fasts with 8-h eating times, 20-h fasts with 4-h feed times, or other similar versions^5^. Time-restricted eating has two variations; eTRE (eating early during the day) and lTRE (eating late during the day). Restricting food intake to the morning seems to result in an improvement of insulin sensitivity, beta-cell responsiveness, blood pressure, inflammation, and oxidative stress. In contrast, on lTRE, restricting food intake to the late afternoon or evening did not change or even worsen blood glucose, beta-cell responsiveness, and lipid levels^6^. TRE has emerged over the past 10 to 15 years as an unconventional and effective approach to potentially reduce body weight and improve metabolic health especially in the elderly population^7^.

Both IF and CR have been found to reduce body weight when delivered overtime periods of four weeks or longer^5^. For example, alternate-day fasting and 5:2 IF regimens have been shown to produce clinically meaningful weight losses similar in magnitude to that of CR. In contrast, TRE regimens typically produce less weight loss than other IF regimens but also preserve muscle mass, both in human and animal models^8^.

The problem of excess body weight applies to all age groups; however, it is most dangerous among older adults, mainly due to the potential to negatively affect metabolic processes and lead to the development of metabolic syndrome^9^. Another crucial issue among older adults is the problem with maintaining an adequate diet. Excess body fat is a factor that may lead to insulin resistance^10^, progressive loss of muscle and bone mass, and cardiovascular disease as well ^11^. Long-term adherence to caloric restriction, especially in elderly is low while adherence intermittent fasting, especially TRE regime sems to be promising^7,12^.

The current study examined the relationship of total abstinence from food intake for 16 h per day, from 08:00 to 12:00 p.m., with body composition and BMI status among overweight, older men. We hypothesized that time-restricted eating (TRE) would be associated with lower BMI, decrease waist circumference and, body fat without changes in muscle mass. Moreover, it was hypothesized that an eating window from 08:00 to 12:00 p.m. would be easy to maintain for early elderly overweight men.

## Materials and methods

### Participants

Forty-six participants were verified for the study and were divided into two groups of 23 participants each. The participants were randomly divided into two groups: an experimental group and a control group (EXP; n = 23; 69.3 ± 2.5 years, height 175 ± 6 cm, body mass 86.3 ± 8.8 kg; CON; n = 23; 69.6 ± 3.3 years, height 176 ± 6 cm, body mass 87.7 ± 8.3 kg).

Inclusion criteria for participation in the experiment:

1. Men, age 65–74 years old.
2. Nonsmoking.
3. BMI; 25–29.9 kg/m^2^.
4. Lack of nutritional disorders.
5. Ability to understand instructions and active participation in the tasks MMSE > 23 p.
6. Agreement to participate in the research and declaration of respect for the IF guidelines.

All participants of the study signed an informed consent form. The study was approved by the Bioethics Committee of the Chamber of Physicians, and the study was conducted in accordance to the guidelines described in the Helsinki Declaration for research involving humans as well. All tests were carried out in the physiological laboratory of the Opole University of Technology.

### Study design, procedures and intervention

The intervention in the EXP group involved entirely abstaining from food for 16 h a day, from 08:00 to 12:00 p.m. (the next day). The task for the control group was to follow an eating plan based on their previous habits. Professional dietician analyzed the weekly, detailed diet consumed by the participants and identified that all participants represented a mixed diet with calorific value suited to their energy demands. The participants were requested to maintain their typical physical activity for the duration of the entire experiment. The data were collected two days before launching the experiment and were repeated two days after completing the six-week diet program.

The following research tools were applied in the study:

- An analysis of body composition and BMI was performed using a SECA mBCA 515 (seca GmbH & Co. KG, Hamburg, Germany) analyzer with eight electrodes.
- The Mini Nutritional Assessment (MNA) was performed on all participants to assess nutritional disorders.
- An assessment of mental state with regard to cognitive abilities was conducted using the Mini–Mental Status Examination (MMSE). The Mini–Mental State Examination, or Folstein test, is a 30-point questionnaire that is used extensively in clinical and research settings to measure cognitive impairment. It is used to estimate the severity and progression of cognitive impairment and to follow the course of cognitive changes in an individual over time. The following four cut-off levels should be employed to classify the severity of cognitive impairment: no cognitive impairment, 24–30; mild cognitive impairment, 19–23; moderate cognitive impairment, 10–18; and severe cognitive impairment, ≤ 9^13^.

Participants from the EXP group were asked to mark in the calendar, which was included in the food diary, days when they were successful with the TRE plan. Data were excluded from the analysis when the number of days without the complete absence of food from 8.00 to 12.00 p.m. (16 h per day) exceeded 10%. All 23 participants in the experimental group completed the six-week program.

### Statistical methods

The collected data were analyzed by application of Jamovi 1.1.9. software. In this study, the authors used ANCOVA to analyze the significance of differences between groups, and the specified Fixed Factor was the GROUP variable, while the Covariates was Baseline variable^14^. It also showed the differences between groups and the 95% confidence intervals for the differences. The sample size of 23 participants in 2 groups is sensitive enough to detect Effect size η^2^_*p*_ = 0.152 power 80% and a 5% (two-sided) significance level. The authors are aware, that there is large effect size, and it requires appropriate interpretation. However, it is important to note, that the study group was specific because it was difficult to find men of this age to participate in a food restriction experiment. Therefore, despite great efforts, only a group of 23 subjects could be included in this experiment. The authors also believe that the final reduction in the number of people in the EXE group did not significantly affect the effect size and its interpretation.

## Results

Table 1 presents the changes in total soft tissue content in the experimental and control groups. The experimental group has higher mean values for each of the significantly different statistical characteristics, as indicated by positive confidence intervals.

**Table 1:** Changes in total soft tissue content in the experimental and control groups.

	EXP		CON		Mean difference EXP/CON	95% CI for mean difference
	Baseline	Follow-up	Baseline	Follow-up		
BMI (kg/m^2^)	28.0 ± 1.65	27.5 ± 1.74	28.38 ± 1.72	28.51 ± 1.63	− 0.62	0.37–0.86
Relative fat mass (%)	28.5 ± 6.92	27.6 ± 5.93	29.82 ± 5.30	30.01 ± 5.24	− 1.29	0.45–2.12
Absolute fat mass (kg)	24.4 ± 6.38	23.4 ± 5.64	26.29 ± 6.20	26.64 ± 6.05	− 1.61	0.80–2.41
Fat-free mass (kg)	62.0 ± 8.90	61.4 ± 8.12	61.16 ± 5.42	61.50 ± 5.46	− 0.70	0.01–1.40
Skeletal muscle mass (kg)	30.7 ± 5.17	30.4 ± 4.76	30.44 ± 3.70	30.19 ± 3.66	− 0.11	− 0.24–0.45
Waist circumference (cm)	97.7 ± 6.21	94.9 ± 5.56	100.26 ± 7.28	100.26 ± 7.23	− 3.11	1.89–4.33
Body weight (kg)	86.3 ± 8.80	84.8 ± 8.79	87.66 ± 8.28	88.07 ± 8.27	− 1.92	1.14–2.70
Visceral fat mass (l)	3.15 ± 1.00	2.60 ± 0.88	3.40 ± 1.11	3.46 ± 1.11	− 0.64	0.46–0.82

After six weeks of TRE, BMI in the EXP group decreased by 0.5 kg/m^2^ with an insignificant increase in the CON group. In the analyzed parameter, the 95% confidence interval for the mean difference (− 0.62) between EXP and CON groups ranges from 0.37 to 0.86.

In both groups, the results of the preliminary measurements showed no difference in body weight (approximately 87 kg) between the groups—while a study conducted after the experiment showed a within-group decrease of 1.5 kg body weight in the experimental group and an increase of 0.41 kg body weight in the control group. The mean difference in body weight between EXP and CON groups after the intervention was − 1.92 kg, with a 95% confidence interval for the mean difference from 1.14 to 2.70. Initial skeletal muscle mass in the EXP group (30.7 ± 5.17 kg) and CON group (30.44 ± 3.7 kg) was not different statistically and, there were no considerable changes after six weeks of the experiment.

Initial waist circumference and visceral fat in the EXP and CON group were not statistically different. After the experiment, waist circumference in the EXP group decreases by almost 3 cm. In the analyzed parameter, the 95% confidence interval for the mean difference (− 3.11) between EXP and CON groups ranges from 1.89 to 4.33. Visceral fat mass decreases in the EXP group by more than 0.5 l without significant changes in CON. For Visceral fat mass, the 95% confidence interval for the mean difference between the EXP and CON groups ranges from 0.01 to 1.40.

Table 2 shows the results of ANCOVA between the two probes of the experimental, and control groups at the two measurement time points. The analyses showed, that only three of the variables analysed were not statistically significant between groups (fat-free mass, skeletal muscle mass and intracellular water). In the analysis performed, the variable BMI was found to have been statistically significant F (1.43) = 25.6, *p* ≤ 0.001. Differences in variables also proved to have been statistically significant: relative fat mass F (1.43) = 9.74, *p* ≤ 0.003, aabsolute fat mass mass F (1.43) = 16.20, *p* ≤ 0.001, *p* ≤ 0.005, waist circumference F (1.43) = 26.30, *p* ≤ 0.001, weight F (1.43) = 24.60, *p* ≤ 0.001 and visceral fat mass F (1.43) = 50.30, *p* ≤ 0.001.

**Table 2:** Results of ANCOVA significance analysis of differences in changes in total soft tissue content between experimental and control groups.

	F (1.43)	*p*	η^2^_*p*_
BMI (kg/m^2^)	25.60	0.001	0.373
Relative fat mass (%)	9.74	0.003	0.185
Absolute fat mass (kg)	16.20	< 0.001	0.273
Fat-free mass (kg)	4.17	0.047	0.088
Skeletal muscle mass (kg)	0.37	0.547	0.008
Waist circumference (cm)	26.30	< 0.001	0.380
Weight (kg)	24.60	< 0.001	0.364
Visceral fat mass (l)	50.30	< 0.001	0.539

Significant values are in bold.

## Discussion

According to the authors’ knowledge, this study is one of the first studies to investigate the impact of TRE on early elderly overweight men. The main achievement of this study was that a six-week TRE intervention was effective in reducing body fat and well-tolerated in early elderly overweight men.

The results of the study are confirmed by the pilot study of Anton et al.^8^ and author’s prior research as well^7^.

After the first few days of fasting, only one participant reported discomfort related to hunger. All 23 (100%) participants succeeded in following the prescribed diet plan, which suggests this eating approach was acceptable and they would like to maintain this type of eating pattern. Thus an emerging body of research suggests TRE is well tolerated by most older adults^12^ and could be a practical strategy for obesity treatment among overweight seniors.

The global life expectancy is constantly increasing. Because the physiology of aging plays a key role in chronic diseases, any strategy that delays the aging process itself would reduce the age-related increase in morbidity and risk of comorbidities^15^. Numerous human studies have confirmed that calorie restriction or increasing fasting time is the only non-genetic intervention that is consistently considered to be the optimal intervention to improve health and life expectancy^16^, increase stress resistance, slow aging, and increase longevity^17^. Moreover, different strategies of fasting or calorie restriction improve homeostasis and protect against cardiovascular, neoplastic, and neurodegenerative diseases^18,19^.

CR is considered to be a very effective anti-aging strategy^20^, however for most people, calorie counting and the inability to eat at will results in progressive frustration, and as a consequence leads to the abandonment of diet plans and a return to old habits, directly leading to a yo-yo effect^15^. Therefore, the prolonged implementation of CR in humans is concerned to be impractical and has poor adherence. CR additionally may trigger several health concerns. Among elderly, these include malnutrition, infertility, bone thinning and osteoporosis, loss of strength and stamina, and psychological conditions such as depression, emotional deadening, and irritability^21^. As a result of the low adherence to prolonged CR and possible adverse changes in metabolism, alternative eating methods have been developed to provide the same benefits^22^. For seniors, diet plans involving the temporary absence of food seem to be one of the most comfortable forms, without the undesirable side effects associated with calorie restriction^23^. Especially time-restricted eating (16/8) seems to be an easy-to-maintain fasting program. TRE has a relatively short fasting period, is well matched to the daily routine, and does not seem to prompt overeating after the periods designed for fasting^7^.

Despite a report of Lowe et al.^24^, which shows that TRE, in the absence of other interventions, is not more effective in weight loss than eating throughout the day, most IF studies have reported reductions in body weight, with results ranging from 2.5% to almost 10% weight loss^25^, depending on the protocol. In accordance with our experiment, after six weeks of TRE, the experimental group lost approximately 2% of body weight, which lead to a 0.5 kg/m2 reduction of BMI in the EXP group without significant changes in body weight in the CON group. The mean decrease from baseline in body weight in the EXP group was − 1.5 kg. The changes in body weight observed in this experiment are analogous to the results of Peeke et al.^26^ (− 2.2 kg) and confirmed by the data from a systematic review of Pellegrini et al.^27^ (− 1.07 kg).

Decreased body weight and BMI in this age group correlated with a lower risk for dementia^28^, cognitive function^29^, and cardiovascular diseases^30^. Participants from the experimental group significantly decreased absolute fat mass (Mean Difference between EXP and CON of − 1.61 kg, 95% CI) and relative fat mass (Mean Difference between EXP and CON of 1.29%, 95% CI) while skeletal muscle mass maintaining almost unchanged. The decrease in fat mass and the improvement of metabolic parameters associated with cardiometabolic health after TRE were also confirmed in studies of Moon et al.^31^.

Reducing fat mass, independent of gender, is directly linked to a lower risk of cardiovascular diseases and osteosarcopenia^32^. Maintain skeletal muscle mass, despite the reduction of body weight and body fat, demonstrates that TRE was not triggering malnutrition and has a positive effect on metabolism as well.

In line with above formulated hypothesis, we also found that TRE was associated with a significant reduction in waist circumference. Many studies have shown that the results of the central obesity measurements have strong correlations with metabolic risk factors^33,34^. Decrease waist circumference in the EXP group would be considered an indicator of health improvement^33,35^.

Our study provides evidence that TRE can produce significant weight loss and improvements in waist circumference. This statement is supported by Xiao et al., who concluded that extended overnight fasting aligned the eating fasting cycle with the internal circadian clock in relatively better way, and additionally both observational and experimental studies have linked longer overnight fasting with lower body weight^36^.

Possibly explanation for these results may be the late-night eating. Eating late is considered to have a destructive effect on human health and body composition. TRE from 8.00 to 12.00 p.m. restricts access to food to the active phase (day) and thereby reduces eating at the “wrong time of the day”^37^.

This research adds support to the growing body of evidence that TRE can improve anthropometric and metabolic parameters in overweight, older adults. Nonetheless, data that supports similar improvements in humans are still relatively sparse. The 16:8 protocol of TRE provides an effective alternative to traditional calorie restriction diets; however, more clinical studies are necessary to understand the effects of TRE on others age groups and changes in the metabolic parameters.

Time-restricted eating in overweight, older men is well tolerated and significantly reduced body fat, waist circumference, and body mass index.

This research adds support to the growing body of evidence that TRE can improve anthropometric and metabolic parameters in overweight, older adults. Our findings suggest that TRE (16/8) could be a practical and effective approach to improve health and combatting the burden of common diseases in early elderly overweight men. Future studies with larger sample sizes and accurate assessment of physical activity could be crucial and thereby taken into consideration.

The research shows that TRE is relatively easy to maintain for early elderly overweight men and may become an essential metabolic syndrome treatment tool in clinical seniors.

## Limitations

Despite the author's efforts, only 23 participants have been recruited to the experiment. This was driven by the Covid virus epidemy and recommendation for this age group to stay at home. In future studies, bigger sample size should be taken into consideration.

## Data Availability

The datasets generated during and/or analyzed during the current study are available from the corresponding author on reasonable request.
